# P-21. Impact of Enteral and Parenteral Nutrition on Mortality and Morbidity in Critically Ill patients with Sepsis: A Systematic Review and Meta Analysis

**DOI:** 10.1093/ofid/ofaf695.252

**Published:** 2026-01-11

**Authors:** Gabriel Cedeno, Jose Matias Zaldumbide, Esteban Marcelo Mogrovejo, Mario S Hinojosa, Erick A Nunez, Pablo César Estrella, Wendy E Luna

**Affiliations:** Universidad San Francisco de Quito, Quito, Pichincha, Ecuador; Universidad San Francisco de Quito, Quito, Pichincha, Ecuador; Universidad San Francisco de Quito, Quito, Pichincha, Ecuador; Universidad Internacional del Ecuador, Quito, Pichincha, Ecuador; Universidad San Francisco de Quito, Quito, Pichincha, Ecuador; Colegio Médico del Perú, Lima, Lima, Peru; NA, Quito, Pichincha, Ecuador

## Abstract

**Background:**

The difference between enteral (EN) and parenteral nutrition (PN) in critical patients with sepsis is crucial for optimizing nutritional support strategies to improve outcomes. Understanding which method offers fewer complications, and improved survival can guide clinical decisions and enhance patient care in intensive care settings.

**Figure:**
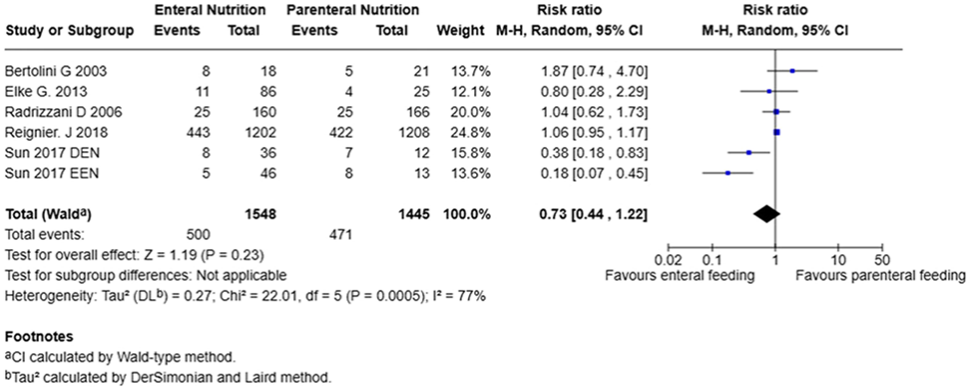
28 Days Mortality Forrest Plot of analysis for 28 days mortality

**Figure:**
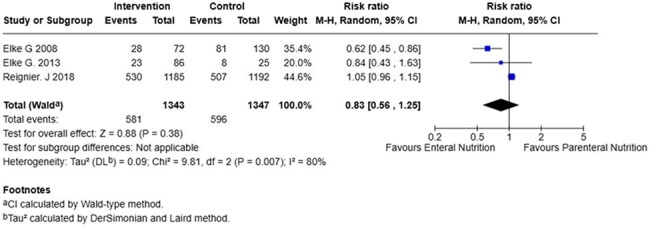
90 Days Mortality Forrest Plot of analysis for 90 days mortality

**Methods:**

PubMed, Scopus and Cochrane databases were searched for randomized controlled trials and observational studies that compare EN to PN in critically ill patients with sepsis and reported the outcomes of (1) 28-day mortality; (2) 90-day mortality; (3) ICU length of stay; and (4) UCI acquired infections. Analysis was made with Cochrane RevMan Version 8.21. A random-effects model was used for outcomes with high heterogenicity and assessed with I^2^ statistics.

**Figure:**
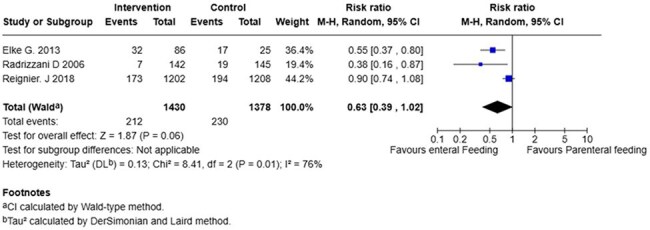
ICU acquired infections Forrest Plot for number of ICU acquired infections

**Figure:**
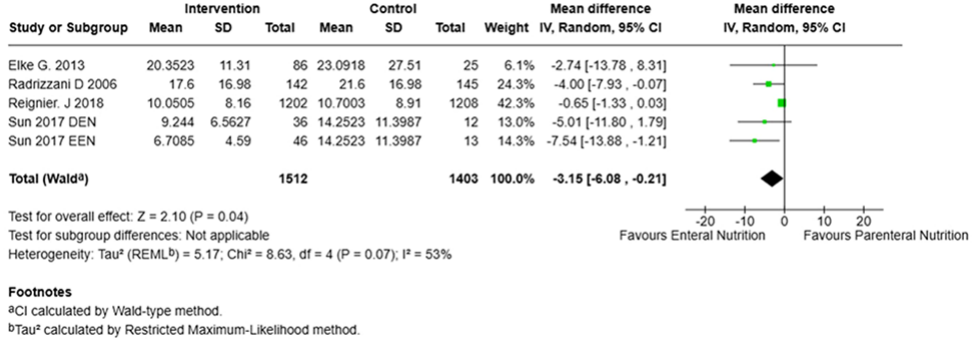
ICU Length of Stay Forrest Plot of Intensive Care Unit Length of Stay

**Results:**

We included 6 studies comprised of 3213 patients, 1628 (50,6%) received EN and 1585 (49.3%) received PN. Mortality at 28-days (RR 0,73; 95% CI 0.44 to 1.22; p=0,23), mortality at 90-days (RR 0.83; 95% CI 0,56 to 1,25; p=0,38) and ICU acquired infections (RR 0,63; 95% CI 0,39-1,02; p=0,06) show no significant difference between the two groups. In contrast, ICU length of stay (mean difference –3,15; 95% CI –6,08 to -0,021; p=0,04) showed a significant reduction in hospital days required for patients with enteral nutrition compared to patients with parenteral nutrition.

**Conclusion:**

While EN was not found to be superior to PN in reducing mortality or ICU-acquired infections in septic critically ill patients, it was associated with a statistically significant reduction in ICU length of stay. These findings support the consideration of EN as a preferred nutritional strategy when feasible.

**Disclosures:**

All Authors: No reported disclosures

